# Ringing a bell in healthcare: harnessing benefits, overcoming implementation challenges, and bridging knowledge gaps of Closed-Loop Oxygen Control systems (CLOCs)

**DOI:** 10.31744/einstein_journal/2024CE0910

**Published:** 2024-03-14

**Authors:** Caroline Gomes Mól, Aléxia Gabriela da Silva Vieira, Raquel Afonso Caserta Eid, Ary Serpa, Marcus J. Schultz, Ricardo Kenji Nawa

**Affiliations:** 1 Hospital Israelita Albert Einstein São Paulo SP Brazil Hospital Israelita Albert Einstein, São Paulo, SP, Brazil.; 2 School of Public Health and Preventive Medicine The Australian and New Zealand Intensive Care Research Centre Monash University Melbourne Australia School of Public Health and Preventive Medicine, The Australian and New Zealand Intensive Care Research Centre, Monash University, Melbourne, Australia.; 3 Austin Health University of Melbourne Melbourne Australia Austin Health, University of Melbourne, Melbourne, Australia.; 4 Amsterdam UMC Amsterdam The Netherlands Amsterdam UMC, Amsterdam, The Netherlands.; 5 MORU Faculty of Tropical Medicine Mahidol University Bangkok Thailand Mahidol Oxford Tropical Medicine Research Unit (MORU), Faculty of Tropical Medicine, Mahidol University, Bangkok, Thailand.; 6 University of Oxford Oxford United Kingdom University of Oxford, Oxford, United Kingdom.; 7 Medical University Vienna Vienna Austria Medical University Vienna, Vienna, Austria.

Dear Editor,

The World Health Organization has listed oxygen as an essential medicine.^(1)^ Commonly prescribed for hospitalized pediatric and adult patients, there is a wide opportunity to improve its effectiveness by promoting the more rational, sustainable, and safe use of supplemental oxygen.^(2,3)^ Guidelines and recommendations for oxygen use may help healthcare workers to adopt and achieve specific oxygen targets,^(4)^ mitigating hyperoxemia, and hypoxemia.^(4,5)^ Staying within safe targets, however, can be challenging, and is also quite time-consuming and labor-intensive, as it requires intensive monitoring and constant manual adjustments of the oxygen flow rate.^(6,7)^ Delays in response time, missing care, and human errors are clear challenges^(8)^ and even short episodes of hypoxemia and hyperoxemia can be critical for specific populations in whom deviations from optimal SpO_2_ ranges can occur often and quickly.

Closed-loop oxygen control systems (CLOCs) have the potential to optimize oxygen flow titration, prevent hypoxemia and hyperoxemia, and reduce oxygen waste.^(6,7)^ With CLOCs, oxygen flow is titrated against continuously monitored SpO_2_ readings and target preset SpO_2_ ranges.^(6,7,9)^ Closed-Loop Oxygen Control systems have already found their way into certain medical devices such as ventilators,^(10)^ low-flow oxygen therapy devices,^(11)^ and high-flow oxygen therapy systems.^(12)^

Closed-Loop Oxygen Control systems not only have the potential to improve patient outcomes,^(6,7)^ but may also reduce the workloads that come with rational, sustainable, and safe use of supplemental oxygen.^(6,7)^ The time saved by CLOCs may further allow healthcare workers to focus on other activities related to patient care, where the presence of healthcare professionals is even more decisive.^(6,12)^ While the safety and efficacy of CLOCs have been extensively studied, evidence of their cost-effectiveness remains to be determined.^(13)^

The demand for oxygen can easily outstrip the supply, and the lack of available oxygen can be a serious problem faced by healthcare systems worldwide, as highlighted recently by the coronavirus disease (COVID-19) pandemic.^(14,15)^ Oxygen scarcity situations can be favorable for CLOCs adoption, as they have a strong potential to reduce waste and optimize the overall usage of supplemental oxygen.^12,1^ An important benefit to be considered in the introduction of CLOCs is their potential to reduce the workload of healthcare professionals, offering a way to partially offload certain responsibilities and help make healthcare systems more efficient.

However, implementing CLOCs in daily care is critical. Although the incorporation of automated technologies at the bedside has demonstrated promising results regarding effectiveness, evidence for patient benefits is probably absent. Another under-discussed topic is the discrepancy and inequitable access to CLOCs devices between high- and middle-income countries. The most updated technologies are frequently available in high-performance organizations. However, this creates an unrealistic scenario for most healthcare institutions worldwide.

Additional studies are required to provide further insights. Effectiveness studies are no longer required once they have been extensively discussed. Closed-Loop Oxygen Control systems perform well and, as expected, outperform healthcare workers. Future studies should focus on cost-effectiveness, workload effects, and implementation in clinical practice to fully examine these topics.
